# Mitochondrial genome comparison and phylogenetic analysis of *Dendrobium* (Orchidaceae) based on whole mitogenomes

**DOI:** 10.1186/s12870-023-04618-9

**Published:** 2023-11-23

**Authors:** Mengting Wang, Wenhui Yu, Jiapeng Yang, Zhenyu Hou, Chao Li, Zhitao Niu, Benhou Zhang, Qingyun Xue, Wei Liu, Xiaoyu Ding

**Affiliations:** 1 College of Life Sciences, Nanjing Normal University, Nanjing, China; 2 Ningbo Key Laboratory of Agricultural Germplasm Resources Mining and Environmental Regulation, College of Science and Technology, Ningbo University, Cixi, China

**Keywords:** Mitochondrial genome, Plastid genome, Phylogeny, *Dendrobium*, Orchidaceae

## Abstract

**Background:**

Mitochondrial genomes are essential for deciphering the unique evolutionary history of seed plants. However, the rules of their extreme variation in genomic size, multi-chromosomal structure, and foreign sequences remain unresolved in most plant lineages, which further hindered the application of mitogenomes in phylogenetic analyses.

**Results:**

Here, we took *Dendrobium* (Orchidaceae) which shows the great divergence of morphology and difficulty in species taxonomy as the study focus. We first de novo assembled two complete mitogenomes of *Dendrobium wilsonii* and *Dendrobium henanense* that were 763,005 bp and 807,551 bp long with multichromosomal structures. To understand the evolution of *Dendrobium* mitogenomes, we compared them with those of four other orchid species. The results showed great variations of repetitive and chloroplast-derived sequences in *Dendrobium* mitogenomes. Moreover, the intergenic content of *Dendrobium* mitogenomes has undergone expansion during evolution. We also newly sequenced mitogenomes of 26 *Dendrobium* species and reconstructed phylogenetic relationships of *Dendrobium* based on genomic mitochondrial and plastid data. The results indicated that the existence of chloroplast-derived sequences made the mitochondrial phylogeny display partial characteristics of the plastid phylogeny. Additionally, the mitochondrial phylogeny provided new insights into the phylogenetic relationships of *Dendrobium* species.

**Conclusions:**

Our study revealed the evolution of *Dendrobium* mitogenomes and the potential of mitogenomes in deciphering phylogenetic relationships at low taxonomic levels.

**Supplementary Information:**

The online version contains supplementary material available at 10.1186/s12870-023-04618-9.

## Background

Mitogenomes of flowering plants displayed unique features with extremely expansive non-coding regions, high recombinations, and frequent sequence transfers, which make them challenging and interesting to study [1]. The extreme variation of plant mitogenomes through inter-species contrasting existed not only between distantly related species but also at family or genus levels. A comparison of six Solanaceae mitochondrial genomes showed huge differences in size (423,596 bp-684,857 bp), similarity (38.13%-55.81%), and gene orders [2]. The mitogenomes of *Silene* undergo experienced unprecedented mutation rate increases and size expansions of more than 40-fold during evolution [3]. Even in closely related species, the structure of mitogenomes still showed various conformations due to intra- or inter-molecular recombination [4]. Understanding the laws of mitogenome variation and evolution at different taxonomic categories was still a challenge in angiosperm. More mitochondrial genome data and comparison analyses of them were needed, especially at low taxonomic levels.

In land plants, mitogenomes usually contained foreign genes or fragments due to horizontal gene transfer (HGT) or intracellular gene transfer (IGT) [5, 6]. Moreover, along with constant mitochondrial genome recombination, such sequence transfer between genomes is continuously ongoing [7]. It was considered one of the potential driving forces for the rapid evolution of mitochondrial genomes with complex compositions and special structures [8]. Furthermore, structural rearrangements and foreign fragment insertion of mitogenomes were closely related to gene chimerism in some plant lineages, resulting in cytoplasmic male sterility which was discovered in *Brassica juncea*, *Oryza sativa*, *Brassica oleracea*, etc. [9–11]. The previous study showed that few HGT events occurred in *Dendrobium* with only one foreign-origin gene [12]. However, to the best of our knowledge, in this genus, there is still a lack of IGT analysis which is also crucial to trace the rearrangements and structural variation of plant mitogenomes.

As one of the significant sources of phylogenetic data, plant mitochondrial genomes were underused in phylogenetic analyses affected by extreme variation, low mutation rates, and difficulty in assembling [13]. In the past few decades, chloroplast sequences have always been used to represent the evolutionary history of cytoplasmic genomes with the assumption of a consistent pattern of inheritance [14]. Indeed, mitochondrial and chloroplast genomes do not necessarily adhere to strict maternal inheritance in angiosperms [15]. The study of McCauley et al. (2013) revealed that the inheritance of the mitochondrial genome has paternal leakage [16], which can break the typically expected linkage equilibrium between cytoplasmic genomes. Such inheritance heterogeneity probably resulted in discordance between the phylogeny of chloroplast genomes and mitochondrial genomes. Therefore, mitochondrial phylogenies are also essential for evolutionary history analyses of flowering plants.

The genus *Dendrobium* (Orchidaceae) with 1200–1500 species spans tropical Asia and Australasia [17]. The *Dendrobium* species are well-known for their morphological diversity with many intergrades and overlapped characteristics [18]. Overlapping distribution and weak reproductive isolation promoted such morphological variation and speciation in the genus [19]. However, the extreme divergence of morphology gave rise to the difficulty of species taxonomy so that phylogenies in some clades were still confused in *Dendrobium* [20]. In particular, mitochondrial well-resolved phylogenetic reconstruction is bare in this genus. Limited sources of mitochondrial genomes (only two high-quality mitogenomes were published in *Dendrobium*) further confine the application of mitogenomes in species taxonomy.

In the present study, we employed Illumina and Nanopore sequence data to assembly mitogenomes of *Dendrobium wilsonii* Rolfe and *Dendrobium henanense* J. L. Lu & L. X. Gao and compared them with other published orchid mitogenomes. Subsequently, we detected sequence transfer from chloroplast genomes to mitochondrial genomes and explored the correlation between IGT, GC content, and repeats. The phylogenies of 26 *Dendrobium* species were constructed based on one plastid and two mitochondrial datasets (whole mitogenomes & mitogenomes excluded chloroplast-derived sequences). The objectives of this study were: (1) to trace variation and evolution of multi-chromosomal structure and genomic content in *Dendrobium* mitogenomes; (2) to assess the frequency of IGT events in *Dendrobium* mitogenomes and the effect of these foreign sequences on phylogenetic construction; (3) to explore the potential of whole mitogenomes in reconstructing phylogenetic relationships at the genus level.

## Results

### Features of *Dendrobium* organellar genomes

**Fig. 1:**
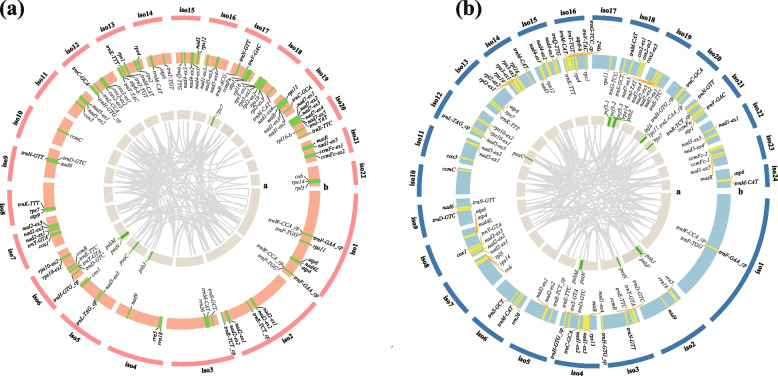
Mitochondrial genome maps of *D*. *wilsonii* (**a**) and *D*. *henanense* (**b**). Isoforms of two mitogenomes are depicted as two circles, respectively. **a** Annotation of plastid-derived genes (only intact genes are shown); (**b**) Mitochondrial gene annotation (grey gene names are reverse genes and black names are forward genes; "-ex" means exon; "-cp" represents tRNAs of plastid origin). Internal curves represent the positions of repeat pairs

Complete mitogenomes of *D*. *wilsonii* and *D*. *henanense* were sequenced and assembled in this study. The mitogenomes were 763,005 bp and 807,551 bp long, displaying atypical multichromosomal structures consisting of 22 and 24 independent isoforms (chromosomes) (Table 1, Fig. 1). The size of each isoform was diversified without a "master circle", ranging from 20,401 bp to 124,954 bp (Additional file 1: Table S1). Most of them were inextensible circular-mapping isoforms, accounting for 87.5% and 86.4% of total isoforms. The GC contents of isoforms varied from 40.2% to 46.2% in *Dendrobium* mitogenomes. Gene contents were counted and compared between *D*. *wilsonii* and *D*. *henanense* mitogenomes. There was a total of 77 and 83 genes annotated. Each isoform contained full-length mitochondrial genes. The numbers of single-copy protein-coding genes were consistent in these two species, with 38 genes. The *atp6* and the *rpl16* both had two different copies in these two mitogenomes. The *cox1* was only doubled in the mitogenome of *D*. *wilsonii*. Three protein-coding genes (*rpl10*, *sdh3*, and *sdh4*) were lost in accordance with other orchid species. A total of 40 tRNA genes (11/40 chloroplast-derived tRNAs) were annotated in the mitogenome of *D. henanense*, whereas* D*. *wilsonii* only contained 33 tRNA genes (9/33 chloroplast-derived tRNAs). Besides, we identified 32 unknown open reading frames (ORFs) in mitochondrial isoforms without protein-coding genes.

**Table 1:** General features of *D*. *wilsonii* and *D*. *henanense* mitogenomes

Genome feature	*D*. *wilsonii*	*D*. *henanense*
Mitogenome length (bp)	763,005	807,551
Gene numbers	77	83
Protein-coding genes	41	40
Single-copy protein-coding genes	38	38
tRNA genes	33	40
rRNA genes	3	3
Repeat content (bp)	165,202 (21.65%)	131,259 (16.25%)
Large repeat	144,026 (18.88%)	105,356 (13.05%)
Intermediate repeat	15,506 (2.03%)	19,229 (2.38%)
Short repeat	5,670 (0.74%)	6,674 (0.83%)
Chloroplast-derived sequence	79,909 (10.5%)	96,511 (12%)
RNA editing sites	571	567

**Fig. 2:**
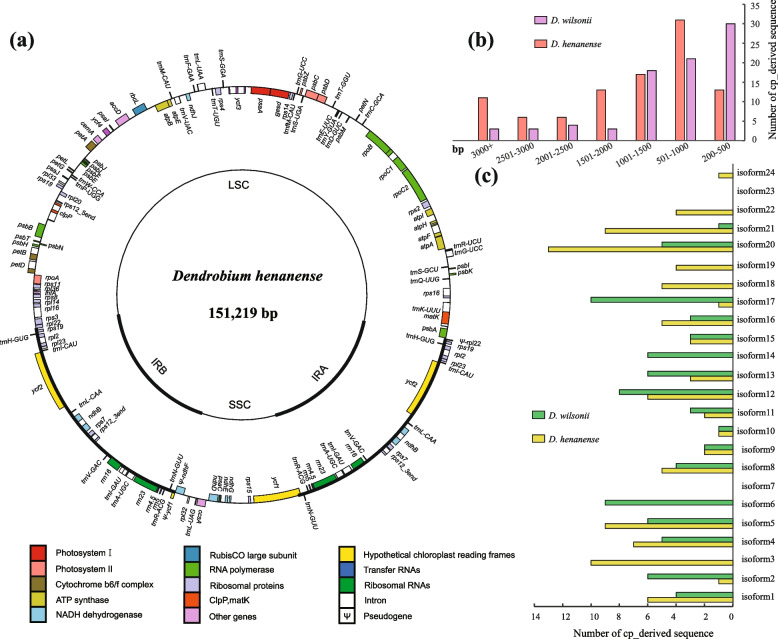
Plastome map of *D*. *henanense*, and plastid-derived sequences in mitogenomes of *D*. *wilsonii* and *D*. *henanense*. **a** Plastome map of *D*. *henanense*. Genes outside the circle are forward and inside the circle are reverse. Different colors represent different functional groups of genes; (**b**) Numbers of plastid-derived sequences with different lengths in mitogenomes of *D*. *wilsonii* and *D*. *henanense*; (**c**) Distributions of plastid-derived sequences in each isoform of *D*. *wilsonii* and *D*. *henanense* mitogenomes

We newly sequenced and assembled the chloroplast genome of *D*. *henanense* into a typical circular structure, consisting of quadripartite regions (two inverted repeats (IR_A_, IR_B_), a large single copy (LSC), and a small single copy (SSC)) (Fig. 2a). The chloroplast genome was 151,219 bp long with 37.52% GC content, while the length of four regions was 26,128 bp (IR), 84,962 bp (LSC), and 14,001 bp (SSC) with 30.35%-43.4% GC contents. The gene contents were well-conserved in *Dendrobium*. A total of 103 genes were annotated, including 69 protein-coding, 30 tRNA, and 4 rRNA genes. Among them, 11 protein-coding genes and 6 tRNA contained introns.

### Codon usage and RNA editing level of mitochondrial genes

The total length of protein-coding genes in *D*. *wilsonii* and *D*. *henanense* were 34,593 bp and 32,730 bp. The start codon was ATG in most of the protein-coding genes, excluding *mttB*. Three typical stop codons (TAA, TGA, and TAG) were detected in all protein-coding genes. The relative synonymous codon usage (RSCU) of all protein-coding genes was calculated using W 1.4.4 (Additional file 2: Table S2). Most NNT or NNA had high RSCU values (> 1.0), such as His (CAU, 1.5/1.48), Gln (CAA, 1.5), and Ser (UCU, 1.43), showing that A or U has a higher percentage at third codons than G or C.

In plant mitogenome, C to U RNA editing was common, playing a significant role in gene expression. We predicted 571 and 567 nonsynonymous editing sites in protein-coding genes of *D*. *wilsonii* and *D*. *henanense* mitogenomes (Table 1). RNA editing sites of the same genes were conservative between these two *Dendrobium* species, except the *ccmFc*, *cob*, and *nad4*. The number of RNA editing sites differed in each gene (Additional file 3: Fig. S1). The *ccmFn* had the most RNA editing sites (40 sites), whereas only two RNA editing sites were discovered in the *rps11*. In addition, editing levels among different codon positions were heterogeneous. The editing levels in second-codon positions were higher than in first-codon and third-codon positions. Notably, no editing sites were detected in the third-codon position of all genes.

### Repeat and SSR analysis

Repeat sequences, including large repeats (> 1000 bp), intermediate repeats (100–1000 bp), and short repeats (< 100 bp), were closely related to recombinational activities and structural variations of plant mitogenomes. A total of 182 and 196 repeats were identified in *D*. *wilsonii* and *D*. *henanense* mitogenomes, which corresponded to 21.65% (165,202 bp) and 16.25% (131,259 bp) of the whole mitogenome length (Table 1). The total lengths of short, intermediate, and large repeats were 5,670 bp (6,674 bp), 15,506 bp (19,229 bp), and 144,026 bp (105,356 bp) in the mitogenome of *D*. *wilsonii* (*D*. *henanense*) (Additional file 4: Fig. S2). In three types of repeats, the number of short repeats accounted for the highest proportion in mitogenomes. The *D*. *wilsonii* mitogenome presents six large repeat pairs, ranging from 8,373 bp to 16,983 bp with high sequence identities (> 99%). Large repeat numbers of *D*. *henanense* (five pairs) were lower compared with *D*. *wilsonii*, ranging from 3,522 to 16,490 bp. The analyses of repeat-mediated recombinational activity showed that a total of 10 and 14 repeat pairs exhibited evidence of recombinational activity (Additional file 5: Fig. S3). These recombinationally active repeats were distributed among isoform 1, 2, 3, 5, 6, 7, 12, 15, and 20 in *D*. *wilsonii* mitogenome (isoform 1, 2, 4, 6, 8, 12, 14, 17, 19, 20, 21, and 24 in *D*. *henanense* mitogenome).

Three types of simple sequence repeats (SSRs) were discovered in mitogenomes, including mononucleotide, dinucleotide, and trinucleotide repeats (Additional file 6: Fig. S4). The total numbers of SSRs were 54 and 62 in mitogenomes of *D*. *wilsonii* and *D*. *henanense*. The distributions of SSRs were diverse in different isoforms. The isoform 1 of *D*. *wilsonii* and isoform 4 of *D*. *henanense* contained the largest number of SSRs (five and nine).

The repetitive content of the chloroplast genome was far less than that of mitogenomes. We only detected 3,542 bp repeats of *D*. *henanense* plastome, including four types ranging from 21 bp-141 bp. Forward, reverse, complement, and palindromic repeats account for 31%, 11%, 2%, and 56% of the total number of repeats (Additional file 7: Fig. S5). The distribution densities of these repeats varied in SSC, LSC, and IR regions. Compared with SSC and IR regions, the LSC region had a higher repeat density. There were 32 SSRs identified in the plastome (Additional file 8: Fig. S6). The numbers of SSRs in LSC, IRs, and SSC regions were 20, 4, and 8 respectively.

### Synteny and gene clusters of two mitogenomes

**Fig. 3:**
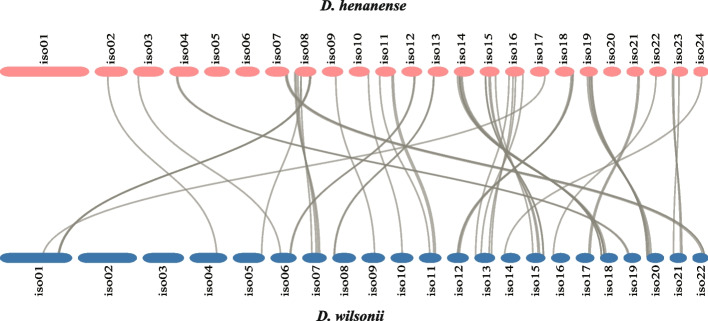
Synteny between mitochondrial genomes of *D*. *wilsonii* and *D*. *henanense*. Syntenic gene pairs between mitogenomes are connected by grey curves

The gene synteny between mitochondrial genomes of *D*. *wilsonii* and *D*. *henanense* were analyzed (Fig. 3). The gene contents of these two mitogenomes were conserved, and most genes are arranged in clusters. But gene orders and positions were various in isoforms. Subsequently, we identified gene clusters of *D*. *wilsonii* and *D*. *henanense* mitogenomes, with two or more adjacent genes (Additional file 9: Table S3). These two mitogenomes shared 14 gene clusters, including *rrn26*-*trnM*-*CAT*, *atp8*-*nad4L*-*atp4*, *trnP*-*TGG- trnW*-*CCA*, *nad2-trnY-GTA*, *trnE-TTC-trnY-GTA*, *atp6_b-trnV-TAC*, *rps3-rpl16-rpl2-rps19*, *atp9-rps7*, *atp6-trnM-CAT*, *atp1-ccmFn*, *rrn5-rrn18*, *nad7-trnI-TAT*, *rps14-rpl5*, and *nad3-rps12*. However, *nad9*-*trnF-GAA* and *trnM*-*CAT*-*trnG*-*GCC* of other *Dendrobium* mitogenomes were absent in these two mitogenomes. Although mitochondrial structures were various due to frequent rearrangements, gene clusters were highly conservative in *D*. *wilsonii* and *D*. *henanense* mitogenomes. These gene clusters were the potential co-transcription units and fragmentation of them probably resulted in partial loss of gene functions. It could explain the relative conservation of gene clusters in mitochondrial genomes.

### Sequence transfer from plastomes to mitogenomes

A total of 79,909 bp and 96,511 bp cp-derived sequences were identified, accounting for 10.5% and 12% of the length of *D*. *wilsonii* and *D*. *henanense* mitogenomes, respectively (Table 1). The length of cp-derived sequences ranged from 216–4,227 bp for *D*. *wilsonii* mitogenome and 263–9,901 bp for *D*. *henanense* mitogenome (Fig. 2b). In *D*. *wilsonii* mitogenome, the range from 200 to 500 bp was most common (30 cp-derived sequences), followed by 501 to 1,000 bp (21 cp-derived sequences). While in *D*. *henanense* mitogenome, the most common length of cp-derived sequences was 501–1,000 bp and followed by 1,001–1,500 bp. There were 5 and 12 intact plastid genes annotated in cp-derived sequences of two mitogenomes (Fig. 1).

To understand the distribution characteristics of transferred sequences, numbers of cp-derived sequences were calculated in different locations of mitogenomes (Fig. 2c). Among different isoforms, cp-derived sequences displayed uneven distribution which was independent of the length of isoforms. For instance, in the mitogenome of *D*. *wilsonii*, most isoforms with cp-derived sequences ranged from 1 to 10, excluding isoform 3, 7, 18, 19, and 22. The isoform 17 had the most cp-derived sequences (10). Similar results were also found in *D*. *henanense* mitogenome, with 1 to 13 cp-derived sequences distributed in 20 of 24 isoforms.

To explore the potential mechanism of continual sequence transferring, we detected the correlation among cp-derived sequences, GC content, and repeats (Additional file 10: Fig. S7). The correlative relationships between cp-derived sequences and GC contents (Pearson’s *r* = -0.34) were higher than cp-derived sequences vs repeats (Pearson’s *r* = -0.07), yet both correlative values were not at a significant level.

### Mitogenome comparison of *D*. *wilsonii* and *D*.* henanense* with other four orchid species

**Fig. 4:**
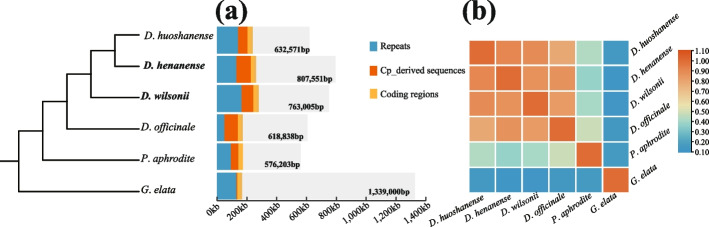
Genomic comparisons among Orchidaceae mitogenomes. **a** Genome size and content of *D*. *wilsonii* and *D*. *henanense* and other four orchid mitogenomes. Lengths of repeats, cp_derived sequences, and coding regions are shown in different colors; (**b**) Similarity among six Orchidaceae mitogenomes. Blue represents low similarity. Red represents relatively high similarity

We compared two newly sequenced mitochondrial genomes with other four orchid mitogenomes (*Dendrobium officinale*: LC640134‐LC640155; *D*. *huoshanense*: LC657527‐LC657545; *Phalaenopsis aphrodite*: MN366132-MN366175; *Gastrodia elata*: MF070084-MF070102). The sizes of mitogenomes vary from 576 kb in *Phalaenopsis aphrodite* to 1,339 kb in *Gastrodia elata* (Fig. 4a). Mitogenomes of *Dendrobium* species and other two orchid species displayed multi-chromosomal structures, consisting of 19–44 isoforms. The contents of protein-coding genes were similar among orchid species, including 37–38 unique genes with 30,969 bp-34,593 bp long.

As expected, sequence similarities at the genus level were higher than at the family level (Fig. 4b, Additional file 11: Fig. S8). *D*. *henanense* mitogenome shared more sequences with *D*. *huoshanense* (91%),* D*. *wilsonii* (86%), and *D*. *officinale* (85%) than other orchid species (*P*. *aphrodite* -37%, *G*. *elata* -11%). Significantly, the mitochondrial genome of *G*. *elata* only shared 11–15% sequences with *Dendrobium* and *Phalaenopsis*, although their phylogenetic relationships were close in orchids. Numerous foreign sequences of *G*. *elata* mitogenome transferred from the mitogenome of its host due to HTG was a potential explanation for such low similarities.

We also examined the contents of repetitive and cp-derived sequences in the Orchidaceae (Fig. 4a). Repetitive sequences were diverse in mitogenomes of these species, ranging from 50,419 bp in *D*. *officinale* to 165,202 bp in *D*. *wilsonii*. Repetitive contents accounted for high proportions of the total length of mitogenomes (8%-22%). The cp-derived content was also an extremely variable feature across the Orchidaceae mitogenomes. There were 6,800 bp-96,511 bp cp-derived sequences identified, accounting for 0.5%-12% of whole mitochondrial sequences. Compared with other orchid species, cp-derived sequences were more abundant in the mitogenomes of *Dendrobium* species.

### Phylogenetic analysis

**Fig. 5:**
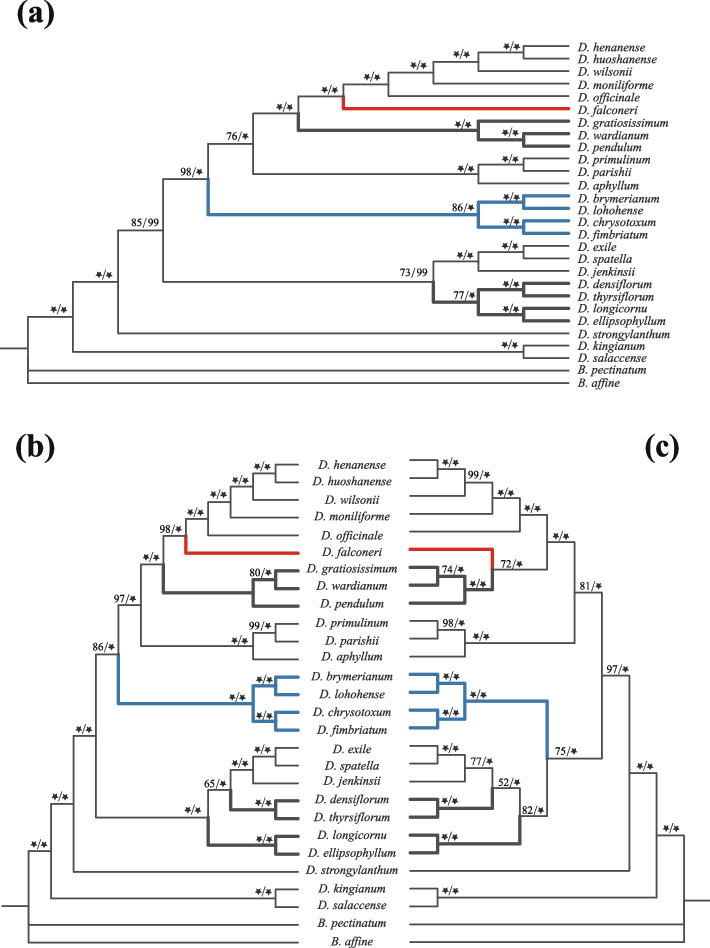
Phylogenies of 26 *Dendrobium* species inferred from whole mitogenomes and plastomes. **a** Plastid phylogeny; (**b**) Mitochondrial phylogeny based on whole mitogenomes; (**c**) Mitochondrial phylogeny based on mitogenomes excluded plastid-derived sequences. Only BI trees of both mitochondrial and plastid phylogenies are shown because topologies in BI trees are almost identical to the results of ML trees (Additional file 1: Fig. S6-S8). The two numbers on each branch were bootstrap supports (BS) of ML analysis and posterior probability (PP) of BI analysis, respectively. Only BS > 50% are shown near the nodes. Black star label BS of 100% or PP of 1.00. Discordances between phylogenies are marked with different colors

In the present study, mitochondrial genomes of 26 *Dendrobium* species were newly assembled, with *D*. *huoshanense* mitogenome as a reference. The phylogenetic relationships of *Dendrobium* were reconstructed based on mitochondrial (matrix 1, matrix 2) and chloroplast genomes (matrix 3) (Fig. 5). Topologies of maximum likelihood (ML) and Bayesian inference (BI) phylogenies displayed high consistency in all three matrices (Additional file 12: Fig. S9 vs Fig. 5a, Additional file 13: Fig. S10 vs Fig. 5b, Additional file 14: Fig. S11 vs Fig. 5c). The backbones of trees based on three matrices were strongly supported with PP > 0.99 and BP_ML_ > 85%, excepting a few nodes.

To understand the effect of foreign sequences of mitogenomes on phylogenetic analyses, the phylogeny of matrix 1 with cp-derived sequences was compared with the matrix 2 (excluded cp-derived sequences) phylogeny (Fig. 5b, 5c). The results showed that the topologies of these two phylogenies were consistent in most clades, excepting two positions: (1) For the phylogeny of matrix 1, *D*. *falconeri* was clustered into the *D*. *officinale* clade comprising monophyly. While in matrix 2, *D*. *falconeri* was the paraphyly with the *D*. *officinale* clade; (2) The phylogenetic position of the *D*. *fimbriatum* clade and *D*. *exile* clade were clustered into monophyly in matrix 2. However, the relationship of these two clades was paraphyly in the phylogeny of matrix 1.

The mitochondrial phylogenies (matrix 1 and matrix 2) were also compared with the chloroplast phylogeny. Phylogenies of mitochondria and plastome were accordant in most clades, except for a few nodes (Fig. 5). The tree of the mitochondrial matrix 1 shared more topological features with the chloroplast tree than the tree of matrix 2. Nevertheless, phylogenetic relationships based on chloroplast and mitochondrial genomes differed in the positions of the *D*. *gratiosissimum* clade and *D*. *densiflorum* clade that immunized the effect of cp-derived sequences. The results showed that the mitochondrial phylogeny displayed unique evolutionary relationships of *Dendrobium*, distinct from the plastid phylogeny. Moreover, the existence of cp-derived sequences would let to an underestimate of the potential inconsistency between the mitochondrial tree and the plastid tree.

## Discussion

### Expansion of mitochondrial genomes in *Dendrobium*

Sizes of mitochondrial genomes have undergone obvious expansion during the evolution of *Dendrobium*. Here, mitochondrial genomes of *D*. *wilsonii* (763 kb) and *D*. *henanense* (807 kb) were larger than that of most orchid species (576 kb-633 kb), except *Gastrodia elata* (1,339 kb) [12, 21, 22]. Coding regions of mitogenomes often make a smaller contribution to flowering plant genome expansions. Although gene loss was often reported in plant mitogenomes [23], the variable length of coding regions accounted for less than 1% of the whole mitochondrial genome. It suggested that enormous intergenic content played a significant role in large sizes of *D*. *wilsonii* and *D*. *henanense* mitogenomes*.* However, mechanisms of massive intergenic content expansion are still unclear. Such expansion was not consistent with the evolutionary relationship, indicating that it was an unstable feature in plant mitochondrial genomes. In this study, the mitogenome size variation of some species in the genus was even larger than that of among genera (e.g., *P*. *Aphrodite* versus *D*. *officinale*; *D*. *henanense* versus *D*. *officinale*). Nevertheless, similar mitogenome sizes did not mean high similarities. Similarities among *Dendrobium* mitogenomes were much higher than that of other orchid species. Significant portions of repetitive and foreign sequences were assumed to drive the intergenic content expansion of mitogenomes [3]. However, our findings showed that mitogenome size and repeats as well as foreign sequences (cp-derived sequences) did not appear to correlate in *Dendrobium*. There are large genomes with fewer repeats or cp-derived sequences and small genomes with many repeats or cp-derived sequences (e.g., *D*. *wilsonii* and versus *D*. *henanense*).

Numerous non-coding regions might have a significant impact on the function of plant mitogenome. Previous studies showed that many isoforms of multi-chromosomal mitogenomes were devoid of intact mitochondrial genes [23], which raises an interesting question about the functional significance and evolutionary roles of these isoforms without mitochondrial genes. Roulet et al., (2020) identified massive unknown open reading frames (ORFs) in 15 isoforms devoid of contact mitochondrial genes, implicating that these isoforms performed some special functions in vivo [5]. One hypothesis involved unknown ORFs in non-coding regions that contributed to cytoplasmic male sterility (CMS) [24, 25]. However, transcriptome analyses of *Brassica oleracea* showed little expression active of most unknown ORFs, which suggested that their special functions are still needed to explore [26]. In our study, mitochondrial genomes of *Dendrobium* also displayed a distinctive multi-chromosomal structure. Although each isoform of mitogenomes contained mitochondrial genes, the density of genes was low in some isoforms with abundant non-coding regions. We also found many ORFs in the non-coding regions, yet whether these ORFs are functional may require further mining of transcriptome data.

### Intracellular sequence transfer from chloroplast genomes to mitochondrial genomes

Sequence exchanges between chloroplast and mitochondrial genomes called intracellular gene transfer (IGT) were common in flowering plants, such as *Zea mays*, *Asclepias syriaca*, and *Magnolia biondii* [27–29]. Frequent homologous recombination events make plant mitogenomes easier to obtain or lose DNA fragments during evolution [30, 31]. The IGT was biased on the sequence transfer of plastomes to mitogenomes because chloroplast genomes are more conserved than mitogenomes in genome content and structure [32–34]. In *Dendrobium*, we found abundant chloroplast-derived fragments of diverse length, which was relatively high compared with other angiosperms (3%-11.5%) [35, 36], indicating frequent IGT existed along with molecular rearrangements in *Dendrobium* species. These cp-derived fragments contained genes with significant functions in the chloroplast, yet, whether they were functional in the mitogenome is unclear in this study. Two possible explanations for the fates of foreign genes in the mtDNA have been proposed: (1) Transferred genes generally lost their functions while the native copies with normal functions coexisted in the mitogenome [8]; (2) Native genes were lost from the mitogenome, and the foreign copies might be functional to maintain the regular operation of cells [37]. Understanding sequence transfer laws are significant to trace ancient recombination events and structural variation in plant mitogenomes, and more attention is needed in this field.

### The effect of foreign sequences of mitogenome on phylogenetic construction in *Dendrobium*

In plants, the foreign sequence is one of the important considerations in phylogeny inferring based on mitochondrial sequences [38]. For mitochondrial genes, some of them were transferred from other angiosperm mitochondrial genomes through HTG. In parasitic plants, the proportions of foreign-derived mitochondrial genes were at a high level, e.g., approximately 33% of mitochondrial genes (12/36) were classified as foreign-origin genes in *Ombrophytum subterraneum* [5]. These foreign-derived genes were typically regarded as interference factors in phylogeny construction and were supposed to be excluded in mitochondrial gene trees inferring. In *Dendrobium*, only *nad1* less than 1000 bp was detected as a foreign origin gene [12], which was almost negligible for whole mitochondrial genomes. Besides, sequence transfer occurred frequently from chloroplast genomes to mitochondrial genomes in angiosperms [29]. Our findings showed that mitochondrial genomes of *Dendrobium* comprised abundant cp-derived sequences as frequent intracellular sequence transfer, accounting for > 10% of lengths of whole mitogenomes (above the average level of angiosperms). Though these foreign sequences were usually excluded directly in phylogeny analyses [38], the description of how these heterologous sequences affect the phylogenetic relationships is still scarce.

To evaluate the effect of sequences with a foreign origin in mitochondrial phylogeny construction, we compared topologies of two mitochondrial trees based on whole mitogenomes (matrix 1) and mitogenomes without cp-derived sequences (matrix 2). Both mitochondrial trees were strongly supported, while their topologies were heterogeneous in a few nodes. The phylogenetic relationships based on mitogenomes with cp-derived sequences showed a part of the features of the plastid phylogeny. The positions of *D*. *falconeri* clade and *D*. *fimbriatum* clade were consistent with the plastid tree. Although these foreign sequences would impact phylogenetic relationships, whether to remove them from mitogenomes depended on specific research objectives. For constructing mitochondrial phylogenies, we suggested removing cp-derived sequences from the mitochondrial genome dataset. To compare the phylogenetic relationships of mitochondrial and plastid trees, we utilized the tree based on the cp-derived sequence excluded dataset as the mitochondrial phylogeny. Nevertheless, mitogenomes with cp-derived sequences can also be a combined dataset of plastomes and mitogenomes, providing more informational loci for species taxonomy and identification, especially in some closely related species.

### The potential of mitochondrial genomes in deciphering phylogenetic relationships at the genus level

Mitochondrial sequences reflect the unique evolutionary history of flowering plants [39]. Plants have three relatively independent genetic materials, including nuclear, chloroplast, and mitochondrial genomes. Generally, nuclear and plastid sequences were applied to phylogenetic analyses [40, 41]. Moreover, because of the limitation of mitochondrial sequences, plastid phylogenies were always used to represent cytoplasmic evolution history [42, 43]. Nevertheless, chloroplast and mitochondrial genomes had different evolutionary histories in some plant groups. Paternal Leakage of mitogenomes or plastomes has been discovered in natural plant populations, which would result in conflicts between organellar phylogenies [15, 44]. In addition, the substitution rate heterogeneity of organellar genomes will impact the phylogenetic relationships of flowering plants [39]. Our study also revealed slight discordances between mitochondrial and plastid phylogenies with high supports. We speculated that it was caused by the evolutionary rate heterogeneity of organellar genomes since organellar phylogenies showed extreme discordances compared with the nuclear phylogeny in our previous study [12]. Together, the above findings suggested that the consideration of mitochondrial phylogenies was complementary rather than redundancy to the present phylogenetic relationship analyses.

The phylogeny based on whole mitochondrial genomes produced new insights into the species taxonomy of *Dendrobium*. Due to the small number (19–41) and low substitution rate of mitochondrial genes, topologies of gene trees were unstable at low taxonomic levels [45, 46]. Phylogenetic positions of several *Dendrobium* species were unresolved or weakly supported in previous analyses based on mitochondrial genes, such as *D*. *falconeri* clade [12]. Here, we reconstructed the phylogeny of *Dendrobium* using whole mitochondrial genome sequences. The topologies of the new mitochondrial phylogeny were consistent with the results of the previous study in most clades. Besides, the weakly supported nodes in the mitochondrial gene tree were well resolved in the present study. For instance, *D*. *falconeri* clade was sister to *D*. *officinale* clade with strong support (100%). Our findings revealed the potential of whole mitochondrial genomes in phylogenetic analyses at low taxonomic levels. Although structures of plant mitogenomes were various with frequent rearrangement, their sequences were relatively conserved at the genus level. In *Dendrobium*, mitogenomes shared far more sequences within the genus (85%-91%) than other orchid species (11%-37%). Furthermore, advances in third-generation sequencing technology provide opportunities for more mitochondrial genomes assembled in many important plant groups [47]. We believe that complete mitogenomes will be applied to the phylogeny or classification in more plant lineages.

## Conclusions

In this study, we explored the evolutionary history of mitochondrial genomes and first applied whole mitochondrial sequences into *Dendrobium* phylogenetic construction. Firstly, we newly assembled two complete mitogenomes of *Dendrobium* and compared them with other orchid mitogenomes, revealing abundant foreign sequences transferred from chloroplast genomes and obvious expansions of non-coding regions in *Dendrobium* mitogenomes. In addition, phylogenetic relationships of 26 *Dendrobium* species were reconstructed based on whole mitochondrial and plastid sequences. Compared to the plastid phylogeny, the phylogenetic relationships of some clades were unique in the mitochondrial tree. Nevertheless, the chloroplast-derived sequences in mitogenomes can narrow the conflict between mitochondrial and plastid trees. For mitochondrial phylogenetic analysis, we recommend considering the effects of these foreign sequences.

## Methods

### Plant materials

Capsules of *Dendrobium wilsonii* and *Dendrobium henanense* were collected from Simao, Yunnan, China (voucher specimen: WMT20211;101°E, 23°N) and Huoshan, Anhui, China (voucher specimen: WMT20212; 116°E, 31°N). Seeds with sterile operations were grown on the MS medium [48]. Root tips were harvested from mature plants for mitochondria extraction. We also sampled 26 *Dendrobium* and two *Bulbophyllum* species for phylogeny analyses and detailed sampling information were listed in Additional file 13: Table S4. All plant materials were authenticated by Prof. Xiaoyu Ding and stored at the greenhouse of Nanjing Normal University.

### DNA isolation and sequencing

To obtain purified mitochondrial DNA (mtDNAs), mitochondria were extracted from 5 g root tips of *D*. *wilsonii* and *D*. *henanense* using improved centrifugation methods [49]. A modified SDS method was applied to isolate mtDNAs [50]. The mitochondrial DNA samples that met the quality requirement (concentration ≥ 20 ng/μl, A260/230 > 1.7, A260/280 = 1.8–2.0) were used to prepare two libraries with 10 kb (Nanopore) and 150 bp (Illumina) respectively (ONT kit: SQK-LSK114). The Nanopore library was loaded to the R10.4.1 chip and sequenced on the PromethION platform (Oxford Nanopore Biosciences, Cambridge, USA). Approximately 77 Gb and 46 Gb long reads were generated. The Illumina library was sequenced on the Illumina Hiseq4000 platform (Illumina, San Diego, USA), generating approximately 10.98 Gb and 10.71 Gb raw short-read data. The long-read data were corrected by Illumina short reads using LoRDEC (kmer value = 19; abundance threshold = 3) [51].

Approximately 0.2 g of fresh leaves of 26 *Dendrobium* and two *Bulbophyllum* species were collected for total DNA extraction using the DNeasy Plant Mini Kit (Qiagen, Hilden, Germany). The total DNA that conformed to the quality requirement (see above) was sonicated to paired-end sequencing using the Illumina Hiseq4000 platform (Illumina, San Diego, USA). We obtained 3–8 Gb 150 bp raw paired-end reads with 450 bp insert size. The raw reads were trimmed by CLC Genomics Workbench 8.5.1 (CLC Biosciences, Aarhus, DK) for removing low-quality reads.

### Mitogenome assembly, and annotation

For the mitochondrial phylogenetic analyses, Illumina paired-end reads of each sample were mapped to the published mitogenomes of *Dendrobium huoshanense* (LC657527‐LC657545) using Genomics Workbench8.5.1. Because plant mitogenomes contained significant amounts of chloroplast-derived sequences through intracellular sequence transfer [6], we deleted all chloroplast-derived sequences from the reference mitogenome to exclude the effects of foreign sequences in phylogeny. Illumina paired-end reads of each sample were also mapped to the reference without transferred plastid DNA using Genomics Workbench8.5.1.

A de novo strategy achieved in SPAdes v3.10.1 (kmer values = 21, 33, 55, 77, 99; phred-offset = 33) [52] was used to assemble mitogenomes of *D*. *wilsonii* and *D*. *henanense* based on Nanopore and Illumina data. A circular assembly would be inferred when a scaffold cannot be extended due to the overlapping between the head and the tail. Illumina data were aligned on the assembly scaffolds from SPAdes v3.10.1 to calculate the read depth using CLC Genomics Workbench8.5.1. Scaffolds with > 20 coverages were selected as candidates of mitochondrial sequences and blasted against the local database consisting of two *Dendrobium* and 46 other angiosperm mitogenomes (Additional file 14: Table S5) using BLASTN (e-value < 1e^−5^). The mitochondrial-related scaffolds were polished through BWA [53] and Pilon [54] to correct the wrong bases and indels again.

For the annotation of mitogenomes, we constructed a local database of angiosperm mitochondrial genes (Additional file 14: Table S5). Mitochondrial sequences were blasted against the database using BLASTN (e-value < 1e^−5^). Exon boundaries and stop and start codons of protein-coding genes were manually corrected using Vector NTI [55]. Genes of tRNA were annotated using tRNAscan-SE 1.21 [56]. Unknown open reading frames (length > 300 bp) were identified using ORF finder (http://www.ncbi.nlm.nih.gov/gorf/orfig.cgi).

### Plastome assembly, and annotation

The chloroplast genome of *D*. *henanense* was assembled using CLC Genomics Workbench8.5.1. The published plastome of *D*. *huoshanense* (LC193517), a closely related species of *D*. *henanense*, served as a reference for assembly. Specific primers of four junction regions (two IRs, LSC, and SSC) were designed to validate the assembly following the procedure of Li et al. (2020) [57]. The assembled plastome was annotated using DOGMA v1.2 [58] and Vector NTI [55]. The stop and start codons of protein-coding genes were manually corrected. Genes of tRNA and rRNA were annotated by tRNAscan-SE 1.21 [56] and rRNAmmer 1.2, respectively (http://www.cbs.dtu.dk/services/RNAmmer/).

### Comparison analyses of mitogenomes

To detect the synteny of mitochondrial genomes of *D*. *wilsonii* and* D*. *henanense*, the MCScanX module of TBtools [59] was applied to map the collinear gene between these two mitogenomes using gene annotation information.

Similarities between mitochondrial genomes were calculated using BLASTN (https://ftp.ncbi.nlm.nih.gov/blast/executables/ blast + /LATEST/) with e-value < 1e^−5^. The redundant sequences were excluded using an automated script. Mauve Build 10 [60] was applied to generate alignments of *Dendrobium* mitogenomes.

### Codon usage, RNA editing level analyses

Codon usage of protein-coding genes was calculated using W 1.4.4 (http://codonw.sourceforge.net). RNA editing sites of protein-coding genes were predicted using PREP-mt with default settings, and duplicated genes were only counted once [61].

### Analyses of repetitive content

Mitochondrial genomes were blasted against themselves for repetitive repeats identified by BLASTN (https://ftp.ncbi.nlm.nih.gov/blast/executables/ blast + /LATEST/) with e-value < 1e^−5^. Repeats with sequence similarity > 95% and sequence length > 20 bp were quantified. Repeats were divided into three types, including short repeats (< 100 bp), intermediate repeats (100–1,000 bp), and large repeats (> 1,000 bp). An automated script was used to delete overlapping sequences among repeat pairs. We also calculated recombination frequencies of repeat pairs based on the method of the previous study [12]. Firstly, we extracted the ± 2,000 bp single-copy flanking regions of each repeat pair as original configurations and built their expected alternative configurations. Then, the long-read data were mapped to these configurations to calculate the number of reads that support each configuration using minimap2 (https://github.com/lh3/minimap2). The recombination frequencies were counted by the formula as follows: reads supported alternative configurations / (reads supported original configurations + reads supported alternative configurations).

Repeats of the chloroplast genome were identified using an online program (REPuter: https://bibiserv.cebitec.uni-bielefeld.de/reputer), including forward, reverse, complement, and palindromic repeats. The Hamming distance was set as three. Only sequence lengths beyond 20 bp were counted.

The MISA (https://webblast.ipkgatersleben.de/misa/) was applied to search simple sequence repeats (SSRs) of mitogenomes and the plastome. The polynucleotide motifs were set as five, and the mononucleotide motifs were set as eight.

### Chloroplast-derived sequence detection

To identify the chloroplast-derived sequences (cp-derived sequence), the mitogenome sequence was blasted against chloroplast genomes of *D*. *wilsonii* (LC490389, downloaded from GenBank) *and D*. *henanense* (LC727398, assembled in this study) using BLASTN with e-value < 1e^−5^. The sequences with identity > 80% and length > 200 bp were defined as chloroplast-derived sequences [2] which were subsequently annotated by Vector NTI.

The correlation between cp-derived sequences, repetitive sequences, and GC contents was detected. Multi-isoforms of *D*. *wilsonii* and* D*. *henanense* mitogenomes were concatenated into a sequence, respectively. Concatenated mitochondrial sequences were cut into 3000 bp bins without overlapping. The numbers of repetitive and cp-derived sequences were counted in each bin. We batch-processed the GC contents of each bin through an automated script. The correlation was evaluated using SPSS Statistics 22.0.

### Phylogeny analyses

Assembled mitochondrial genomes of 26 *Dendrobium* species and two *Bulbophyllum* species with multi-isoforms were much longer than chloroplast genomes. For this reason, we first aligned mitochondrial sequences of each isoform by MAFFTv7 [62] to reduce the computation. Aligned isoforms were concatenated as the mitochondrial matrix 1. Mitochondrial genomes excluded chloroplast-derived sequences were also aligned in the same way, constituting the mitochondrial matrix 2. Chloroplast genomes of 26 *Dendrobium* and two *Bulbophyllum* species were downloaded from the NCBI (https://www.ncbi.nlm.nih.gov) and aligned by MAFFT v7 [62] as matrix 3 (GenBank accession numbers were listed in Additional file 13: Table S4). Alignments with more than 50% missing data were removed. *Bulbophyllum pectinatum* and *Bulbophyllum affine* were set as outgroups.

The ML phylogenies of mitochondrial and plastome matrices were constructed by RAxML v8.0.0 [63] using GTRGAMMA model and 1000 bootstrap replicates. The BI phylogenies were constructed by MrBayes v3.2.5 [64] with the best-fit model estimated by ModelTest v3.7 [65]. Two independent Markov Chain Monte Carlo (MCMC) chains were operated for 5,000,000 generations, and every 1,000 generations were sampled one tree. The first 25% of the sampled trees were burned. The topology of mitochondrial and plastome trees was checked by FigTree v1.4.2 (http://tree.bio.ed.ac.uk/software/figtree/).

### Supplementary Information

**Additional file 1:** **Table S1.** Isoform features of *D*.* wilsonii* and *D*. *henanense* mitogenomes.

**Additional file 2:** **Table S2.** Relative synonymous codon usage of mitochondrial protein-coding genes in *D*. *wilsonii *and* D. henanense *mitogenomes.

**Additional file 3:** **Figure S1.** The number of RNA editing sites of 38 protein-coding genes in mitogenomes of *D*. *wilsonii* and *D*. *henanense*. Blue indicates the number of editing sites at first-codon positions, red at second positions, and grey at third positions.

**Additional file 4:** **Figure S2.** Distributions of repeats in mitogenomes of *D*. *wilsonii* and *D*. *henanense*.

**Additional file 5:** **Figure S3.** Repeat-mediated recombinational activity in mitogenomes of *D*. *wilsonii* and *D*. *henanense*.

**Additional file 6:** **Figure S4.** Distributions of SSRs in different isoforms of *D*. *wilsonii* and *D*. *henanense* mitogenomes. Three types of SSRs are marked with different colors.

**Additional file 7:** **Figure S5.** Four types of repeats in *D*. *henanense *plastome.

**Additional file 8:** **Figure S6.** Distributions of SSRs in *D*. *henanense *plastome. Different types of SSRs are marked with different colors.

**Additional file 9:** **Table S3.** Gene clusters of two *Dendrobium* mitogenomes.

**Additional file 10: Figure S7.** Numbers of repeats, cp_deived sequences, and GC contents in nonoverlapping bins of 3000 bp each through the mitogenomes of *D*. *wilsonii* and *D*. *henanense*.

**Additional file 11:** **Figure S8.** Mauve alignments of *Dendrobium* mitogenomes. The mitogenome of *D*. *huoshanense* was selected as reference. Red lines represent boundaries between isoforms, the order was consistent with the genomic map of mitogenomes.

**Additional file 12:** **Figure S9.** ML tree of 26 *Dendrobium* species inferred from whole plastomes. The number at each node was the percentage of bootstrap value (BS >50% was shown).

**Additional file 13:** **Figure S10.** ML tree of 26 *Dendrobium* species inferred from whole mitogenomes. The number at each node was the percentage of bootstrap value (BS >50% was shown).

**Additional file 14:** **Figure S11.** ML tree of 26 *Dendrobium* species inferred from whole mitogenomes excluded plastid-derived sequences. The number at each node was the percentage of bootstrap value (BS >50% was shown).

**Additional file 15:** **Table S4.** Sampling information of 28 species including two outgroups and 26 *Dendrobium* species.

**Additional file 16:** **Table S5.** Accession numbers of 48 angiosperm mitogenomes for local database establishment.

## Data Availability

The raw sequence data used in this study were deposited in NCBI under the BioProject accession number PRJNA933365 (https://www.ncbi.nlm.nih.gov/bioproject/PRJNA933365). The assembled mitochondrial genome sequences were submitted to the DDBJ with accession numbers LC744518-LC744539 and LC744540-LC744563 (http://getentry.ddbj.nig.ac.jp). The phylogenomic matrices were deposited in the Treebase (http://www.treebase.org, http://purl.org/phylo/treebase/phylows/study/TB2:S29956?x-access-code=bdf7b5197989c5d54f4ab3eccc0194eb&format=html).

## Abbreviations

HGT: Horizontal Gene Transfer
IGT: Intracellular Gene Transfer
mtDNA: Mitochondrial DNA
Cp: Chloroplast
LSC: Large Single Copy
SSC: Small Single Copy
IR: Inverted Repeat
SSRs: Simple Sequence Repeats
ORFs: Open Reading Frames
RSCU: Synonymous Codon Usage
ML: Maximum Likelihood
BI: Bayesian Inference
CMS: Cytoplasmic male sterility
