# Supplementary material for: Mitochondrial genome comparison and phylogenetic analysis of Dendrobium (Orchidaceae) based on whole mitogenomes
Source: BMC Plant Biol. 2023 Nov 23;23:586. doi: 10.1186/s12870-023-04618-9 (PMC10666434; doi:10.1186/s12870-023-04618-9)
Supplement: Supplementary file 1 — Additional file 1: Table S1. Isoform features of D. wilsonii and D. henanense mitogenomes. [file 12870_2023_4618_MOESM1_ESM.docx]

Table S1. Isoform features of *D*. *wilsonii* and *D*. *henanense* mitogenomes.

| Species | Isoform | Length(bp) | Form | GC(%) |
| --- | --- | --- | --- | --- |
| *D*. *wilsonii* | isoform1 | 94,044 | linear | 43.84 |
|  | isoform2 | 76,793 | linear | 42.65 |
|  | isoform3 | 53,641 | circular | 43.57 |
|  | isoform4 | 48,644 | circular | 42.50 |
|  | isoform5 | 41,508 | linear | 41.90 |
|  | isoform6 | 33,751 | circular | 43.66 |
|  | isoform7 | 31,628 | circular | 44.22 |
|  | isoform8 | 30,433 | circular | 43.05 |
|  | isoform9 | 30,227 | circular | 44.04 |
|  | isoform10 | 29,440 | circular | 44.29 |
|  | isoform11 | 28,926 | circular | 43.77 |
|  | isoform12 | 28,064 | circular | 44.21 |
|  | isoform13 | 26,656 | circular | 40.47 |
|  | isoform14 | 25,322 | circular | 44.18 |
|  | isoform15 | 25,036 | circular | 46.12 |
|  | isoform16 | 24,507 | circular | 43.02 |
|  | isoform17 | 24,110 | circular | 44.94 |
|  | isoform18 | 22,956 | circular | 46.09 |
|  | isoform19 | 22,484 | circular | 45.45 |
|  | isoform20 | 22,421 | circular | 44.16 |
|  | isoform21 | 22,013 | circular | 46.10 |
|  | isoform22 | 20,401 | circular | 44.64 |
|  |  |  |  |  |
| *D*. *henanense* | isoform1 | 124,954 | linear | 41.84 |
|  | isoform2 | 45,997 | circular | 42.63 |
|  | isoform3 | 42,507 | circular | 41.68 |
|  | isoform4 | 40,807 | circular | 43.62 |
|  | isoform5 | 35,580 | circular | 41.42 |
|  | isoform6 | 33,762 | circular | 43.47 |
|  | isoform7 | 33,473 | circular | 43.45 |
|  | isoform8 | 29,987 | circular | 44.00 |
|  | isoform9 | 29,649 | circular | 43.99 |
|  | isoform10 | 29,016 | circular | 44.13 |
|  | isoform11 | 28,720 | circular | 43.61 |
|  | isoform12 | 28,693 | circular | 43.22 |
|  | isoform13 | 28,625 | circular | 42.75 |
|  | isoform14 | 28,227 | circular | 45.11 |
|  | isoform15 | 27,483 | circular | 45.94 |
|  | isoform16 | 26,911 | circular | 40.24 |
|  | isoform17 | 26,906 | circular | 42.68 |
|  | isoform18 | 26,760 | circular | 42.88 |
|  | isoform19 | 24,847 | linear | 44.78 |
|  | isoform20 | 24,517 | circular | 40.79 |
|  | isoform21 | 23,941 | circular | 45.08 |
|  | isoform22 | 23,825 | circular | 43.14 |
|  | isoform23 | 21,847 | circular | 46.27 |
|  | isoform24 | 20,517 | linear | 45.03 |
