# Supplementary material for: Mitochondrial genome comparison and phylogenetic analysis of Dendrobium (Orchidaceae) based on whole mitogenomes
Source: BMC Plant Biol. 2023 Nov 23;23:586. doi: 10.1186/s12870-023-04618-9 (PMC10666434; doi:10.1186/s12870-023-04618-9)
Supplement: Supplementary file 2 — Additional file 2: Table S2. Relative synonymous codon usage of mitochondrial protein-coding genes in D. wilsonii and D. henanense mitogenomes. [file 12870_2023_4618_MOESM2_ESM.docx]

Table S2. Relative synonymous codon usage of mitochondrial protein-coding genes in *D*. *wilsonii* and *D. henanense* mitogenomes.

| Species | Amino acid | Codon | Count | RSCU | Amino acid | Codon | Count | RSCU |
| --- | --- | --- | --- | --- | --- | --- | --- | --- |
| *D*. *wilsonii* | Phe | UUU | 397 | 1.07 | Ser | UCU | 259 | 1.43 |
|  |  | UUC | 344 | 0.93 |  | UCC | 168 | 0.93 |
|  | Leu | UUA | 221 | 1.17 |  | UCA | 223 | 1.23 |
|  |  | UUG | 236 | 1.25 |  | UCG | 148 | 0.82 |
|  |  | CUU | 243 | 1.29 | Pro | CCU | 234 | 1.43 |
|  |  | CUC | 121 | 0.64 |  | CCC | 136 | 0.83 |
|  |  | CUA | 185 | 0.98 |  | CCA | 188 | 1.15 |
|  |  | CUG | 123 | 0.65 |  | CCG | 96 | 0.59 |
|  | Ile | AUU | 356 | 1.25 | Thr | ACU | 207 | 1.36 |
|  |  | AUC | 261 | 0.92 |  | ACC | 136 | 0.89 |
|  |  | AUA | 234 | 0.82 |  | ACA | 174 | 1.14 |
|  | Met | AUG | 327 | 1 |  | ACG | 93 | 0.61 |
|  | Val | GUU | 218 | 1.22 | Ala | GCU | 303 | 1.64 |
|  |  | GUC | 137 | 0.77 |  | GCC | 155 | 0.84 |
|  |  | GUA | 199 | 1.11 |  | GCA | 189 | 1.02 |
|  |  | GUG | 161 | 0.9 |  | GCG | 92 | 0.5 |
|  | Tyr | UAU | 224 | 1.43 | Cys | UGU | 84 | 1.1 |
|  |  | UAC | 89 | 0.57 |  | UGC | 69 | 0.9 |
|  | TER | UAA | 15 | 1.1 | TER | UGA | 13 | 0.95 |
|  |  | UAG | 13 | 0.95 | Trp | UGG | 162 | 1 |
|  | His | CAU | 232 | 1.5 | Arg | CGU | 172 | 1.22 |
|  |  | CAC | 77 | 0.5 |  | CGC | 85 | 0.6 |
|  | Gln | CAA | 255 | 1.5 |  | CGA | 176 | 1.25 |
|  |  | CAG | 85 | 0.5 |  | CGG | 111 | 0.79 |
|  | Asn | AAU | 220 | 1.31 | Ser | AGU | 179 | 0.99 |
|  |  | AAC | 116 | 0.69 |  | AGC | 110 | 0.61 |
|  | Lys | AAA | 293 | 1.16 | Arg | AGA | 200 | 1.42 |
|  |  | AAG | 213 | 0.84 |  | AGG | 102 | 0.72 |
|  | Asp | GAU | 251 | 1.34 | Gly | GGU | 268 | 1.29 |
|  |  | GAC | 125 | 0.66 |  | GGC | 111 | 0.54 |
|  | Glu | GAA | 321 | 1.37 |  | GGA | 297 | 1.43 |
|  |  | GAG | 147 | 0.63 |  | GGG | 152 | 0.73 |
|  |  |  |  |  |  |  |  |  |
| *D*. *henanense* | Phe | UUU | 371 | 1.07 | Ser | UCU | 247 | 1.43 |
|  |  | UUC | 323 | 0.93 |  | UCC | 158 | 0.92 |
|  | Leu | UUA | 208 | 1.16 |  | UCA | 215 | 1.25 |
|  |  | UUG | 234 | 1.3 |  | UCG | 142 | 0.82 |
|  |  | CUU | 232 | 1.29 | Pro | CCU | 221 | 1.44 |
|  |  | CUC | 115 | 0.64 |  | CCC | 127 | 0.83 |
|  |  | CUA | 176 | 0.98 |  | CCA | 177 | 1.16 |
|  |  | CUG | 115 | 0.64 |  | CCG | 87 | 0.57 |
|  | Ile | AUU | 341 | 1.27 | Thr | ACU | 194 | 1.37 |
|  |  | AUC | 242 | 0.9 |  | ACC | 126 | 0.89 |
|  |  | AUA | 221 | 0.82 |  | ACA | 157 | 1.11 |
|  | Met | AUG | 299 | 1 |  | ACG | 90 | 0.63 |
|  | Val | GUU | 202 | 1.21 | Ala | GCU | 281 | 1.61 |
|  |  | GUC | 130 | 0.78 |  | GCC | 149 | 0.86 |
|  |  | GUA | 189 | 1.13 |  | GCA | 176 | 1.01 |
|  |  | GUG | 149 | 0.89 |  | GCG | 90 | 0.52 |
|  | Tyr | UAU | 207 | 1.42 | Cys | UGU | 84 | 1.12 |
|  |  | UAC | 85 | 0.58 |  | UGC | 66 | 0.88 |
|  | TER | UAA | 14 | 1.08 | TER | UGA | 12 | 0.92 |
|  |  | UAG | 13 | 1 | Trp | UGG | 149 | 1 |
|  | His | CAU | 214 | 1.48 | Arg | CGU | 162 | 1.2 |
|  |  | CAC | 75 | 0.52 |  | CGC | 83 | 0.61 |
|  | Gln | CAA | 248 | 1.5 |  | CGA | 169 | 1.25 |
|  |  | CAG | 82 | 0.5 |  | CGG | 106 | 0.78 |
|  | Asn | AAU | 209 | 1.3 | Ser | AGU | 168 | 0.97 |
|  |  | AAC | 113 | 0.7 |  | AGC | 105 | 0.61 |
|  | Lys | AAA | 286 | 1.16 | Arg | AGA | 191 | 1.41 |
|  |  | AAG | 206 | 0.84 |  | AGG | 102 | 0.75 |
|  | Asp | GAU | 239 | 1.33 | Gly | GGU | 248 | 1.3 |
|  |  | GAC | 121 | 0.67 |  | GGC | 101 | 0.53 |
|  | Glu | GAA | 310 | 1.37 |  | GGA | 275 | 1.44 |
|  |  | GAG | 144 | 0.63 |  | GGG | 139 | 0.73 |
