# Supplementary material for: Mitochondrial genome comparison and phylogenetic analysis of Dendrobium (Orchidaceae) based on whole mitogenomes
Source: BMC Plant Biol. 2023 Nov 23;23:586. doi: 10.1186/s12870-023-04618-9 (PMC10666434; doi:10.1186/s12870-023-04618-9)
Supplement: Supplementary file 3 — Additional file 3: Figure S1. The number of RNA editing sites of 38 protein-coding genes in mitogenomes of D. wilsonii and D. henanense. Blue indicates the number of editing sites at first-codon positions, red at second positions, and grey at third positions. [file 12870_2023_4618_MOESM3_ESM.docx]

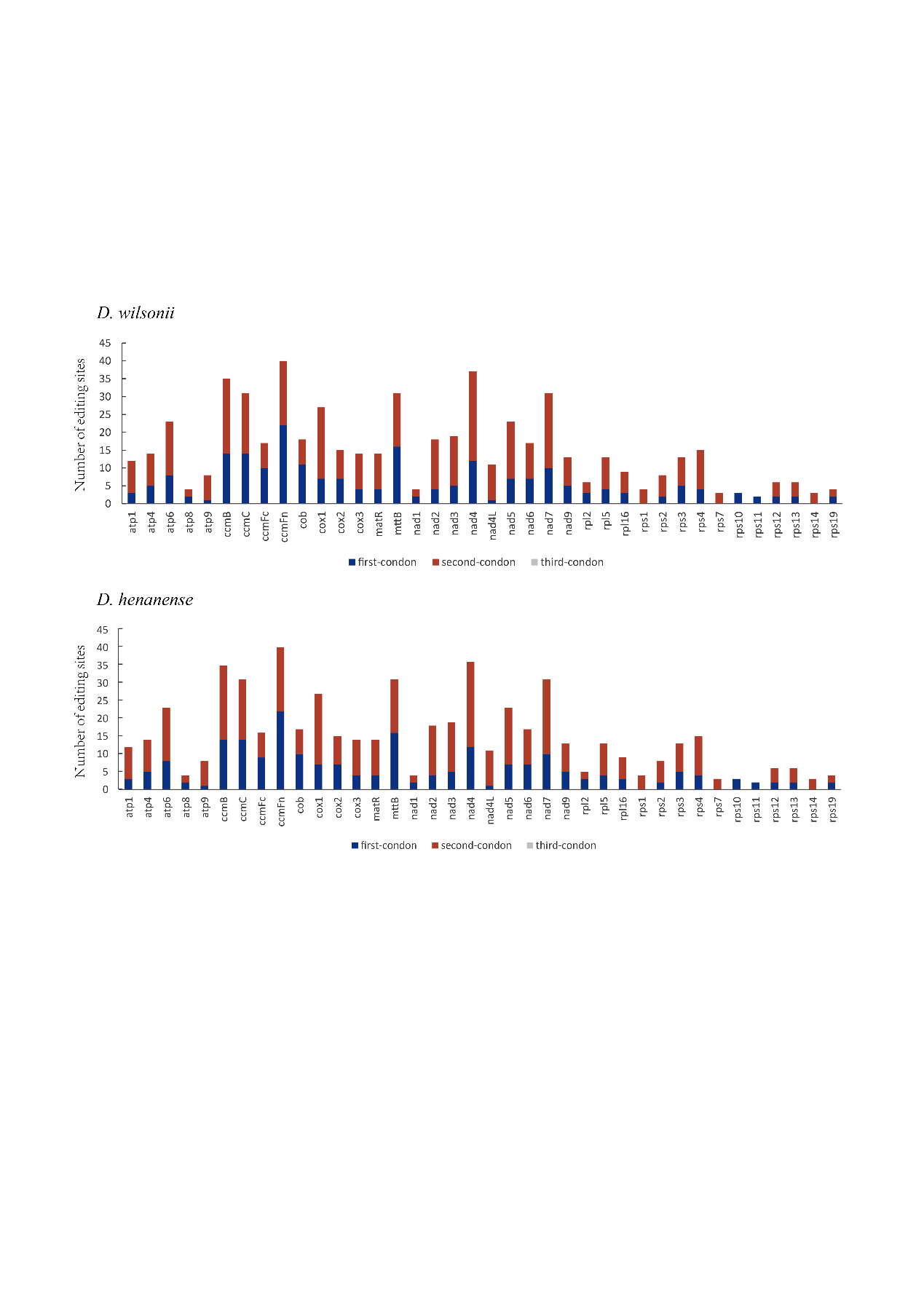


**Additional file 3: Figure S1.** The number of RNA editing sites of 38 protein-coding genes in mitogenomes of *D*. *wilsonii* and *D*. *henanense*. Blue indicates the number of editing sites at first-codon positions, red at second positions, and grey at third positions.
