# Supplementary material for: Mitochondrial genome comparison and phylogenetic analysis of Dendrobium (Orchidaceae) based on whole mitogenomes
Source: BMC Plant Biol. 2023 Nov 23;23:586. doi: 10.1186/s12870-023-04618-9 (PMC10666434; doi:10.1186/s12870-023-04618-9)
Supplement: Supplementary file 4 — Additional file 4: Figure S2. Distributions of repeats in mitogenomes of D. wilsonii and D. henanense. [file 12870_2023_4618_MOESM4_ESM.docx]

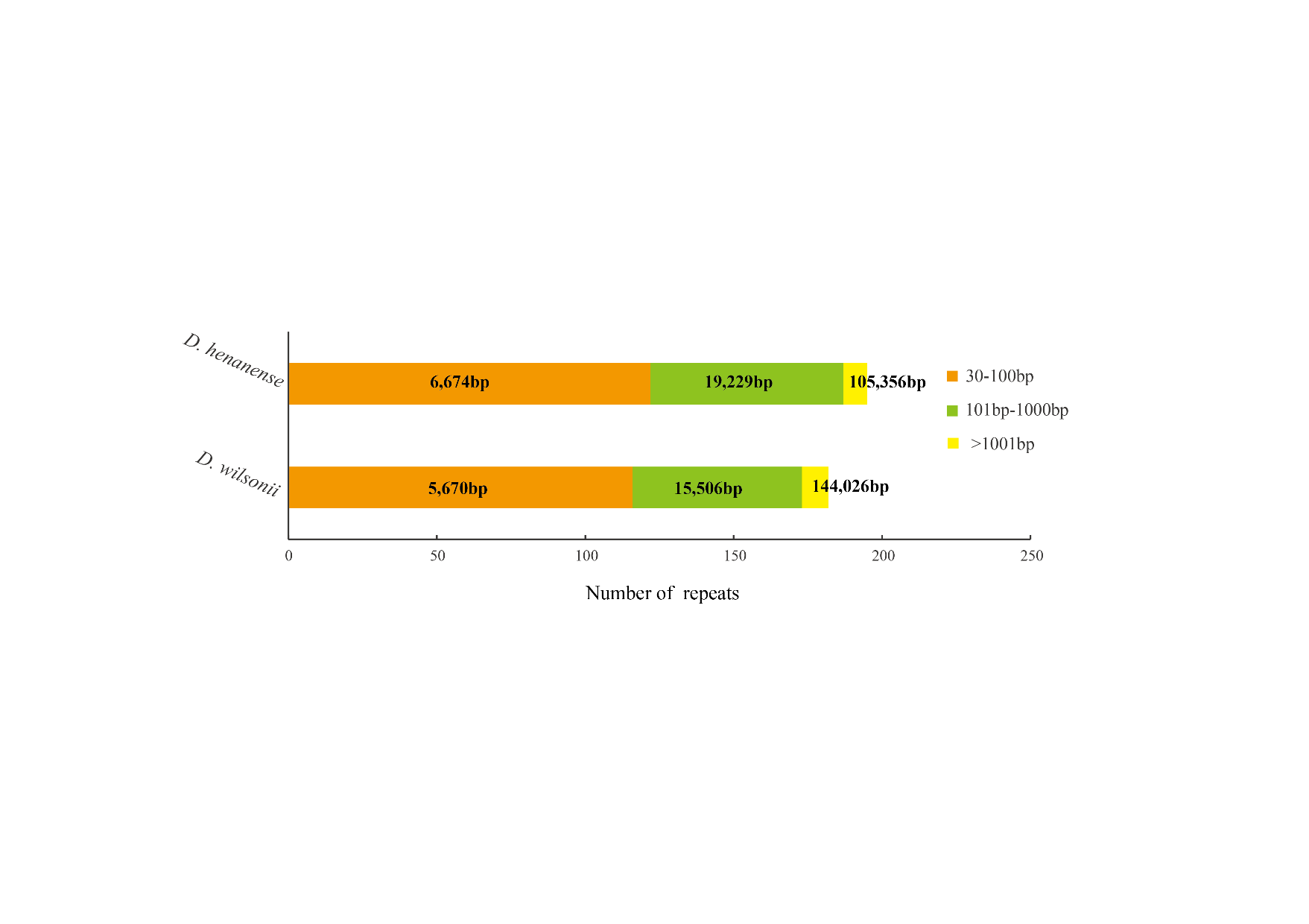


**Additional file 4: Figure S2.** Distributions of repeats in mitogenomes of *D*. *wilsonii* and *D*. *henanense*.
