# Supplementary figures and images for: Mitochondrial genome comparison and phylogenetic analysis of Dendrobium (Orchidaceae) based on whole mitogenomes
Source: BMC Plant Biol. 2023 Nov 23;23:586. doi: 10.1186/s12870-023-04618-9 (PMC10666434; doi:10.1186/s12870-023-04618-9)

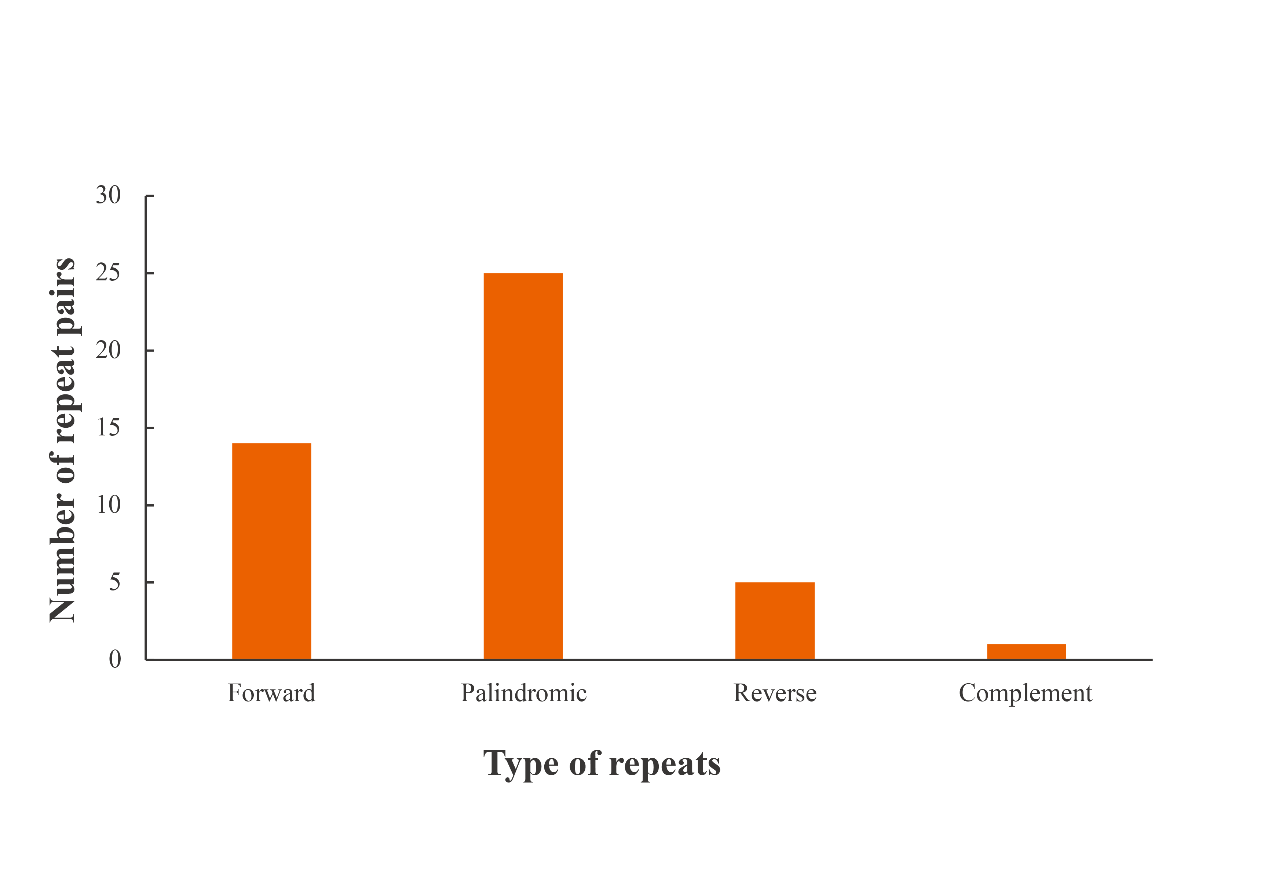


**Additional file 7: Figure S5.** Four types of repeats in *D*. *henanense* plastome.

Supplement: Supplementary file 7 — Additional file 7: Figure S5. Four types of repeats in D. henanense plastome. [file 12870_2023_4618_MOESM7_ESM.docx]
