# Supplementary material for: Mitochondrial genome comparison and phylogenetic analysis of Dendrobium (Orchidaceae) based on whole mitogenomes
Source: BMC Plant Biol. 2023 Nov 23;23:586. doi: 10.1186/s12870-023-04618-9 (PMC10666434; doi:10.1186/s12870-023-04618-9)
Supplement: Supplementary file 8 — Additional file 8: Figure S6. Distributions of SSRs in D. henanense plastome. Different types of SSRs are marked with different colors. [file 12870_2023_4618_MOESM8_ESM.docx]

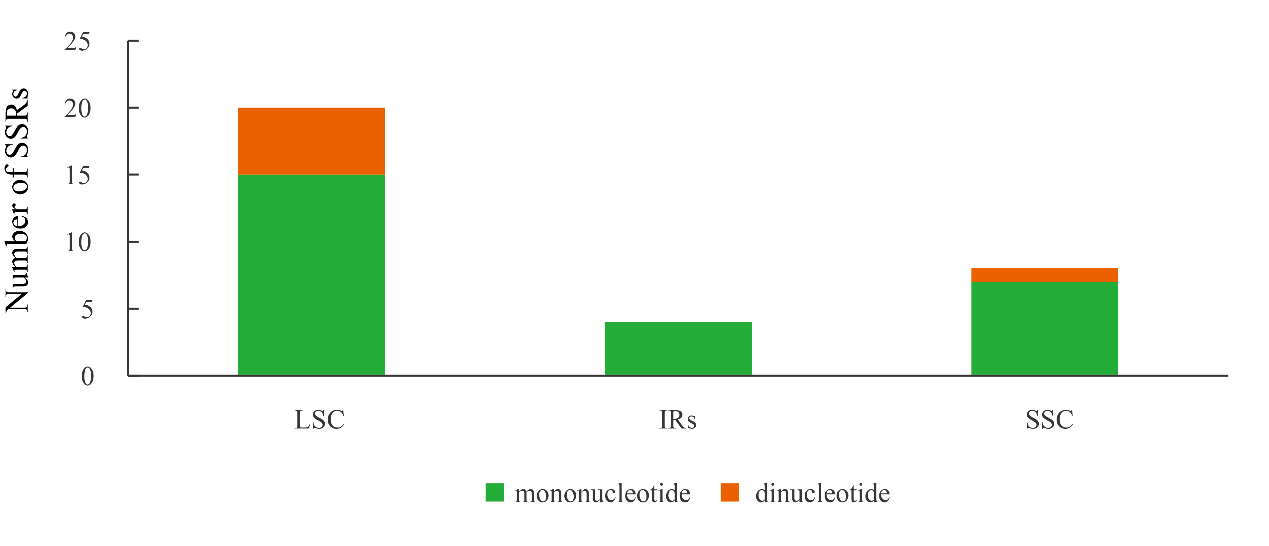


**Additional file 8: Figure S6.** Distributions of SSRs in *D*. *henanense* plastome. Different types of SSRs are marked with different colors.
