# Supplementary material for: Mitochondrial genome comparison and phylogenetic analysis of Dendrobium (Orchidaceae) based on whole mitogenomes
Source: BMC Plant Biol. 2023 Nov 23;23:586. doi: 10.1186/s12870-023-04618-9 (PMC10666434; doi:10.1186/s12870-023-04618-9)
Supplement: Supplementary file 9 — Additional file 9: Table S3. Gene clusters of two Dendrobium mitogenomes. [file 12870_2023_4618_MOESM9_ESM.docx]

Table S3. Gene clusters of two *Dendrobium* mitogenomes.

| Gene cluster | *D. wilsonii* | *D. henanense* |
| --- | --- | --- |
| *rrn26*-*trnM-CAT* | **+** | **+** |
| *atp8*-*nad4L*-*atp4* | **+** | **+** |
| *nad9*-*trnF-GAA* | **-** | **-** |
| *trnP-TGG*-*trnW-CCA* | **+** | **+** |
| *nad2*-*trnY-GTA* | **+** | **+** |
| *trnE-TTC*-*trnY-GTA* | **+** | **+** |
| *trnM-CAT*-*trnG-GCC* | **-** | **-** |
| *atp6*_b-*trnV-TAC* | **+** | **+** |
| *rps3*-*rpl16*-*rpl2*-*rps19* | **+** | **+** |
| *atp9*-*rps7* | **+** | **+** |
| *atp6*-*trnM-CAT* | **+** | **+** |
| *atp1*-*ccmFn* | **+** | **+** |
| *rrn5*-*rrn18* | **+** | **+** |
| *nad7*-*trnI-TAT* | **+** | **+** |
| *rps14*-*rpl5* | **+** | **+** |
| *nad3*-*rps12* | **+** | **+** |

"+" represents the existence of a gene cluster;

"-" represents the absence of a gene cluster.
