# Supplementary material for: Mitochondrial genome comparison and phylogenetic analysis of Dendrobium (Orchidaceae) based on whole mitogenomes
Source: BMC Plant Biol. 2023 Nov 23;23:586. doi: 10.1186/s12870-023-04618-9 (PMC10666434; doi:10.1186/s12870-023-04618-9)
Supplement: Supplementary file 11 — Additional file 11: Figure S8. Mauve alignments of Dendrobium mitogenomes. The mitogenome of D. huoshanense was selected as reference. Red lines represent boundaries between isoforms, the order was consistent with the genomic map of mitogenomes. [file 12870_2023_4618_MOESM11_ESM.docx]

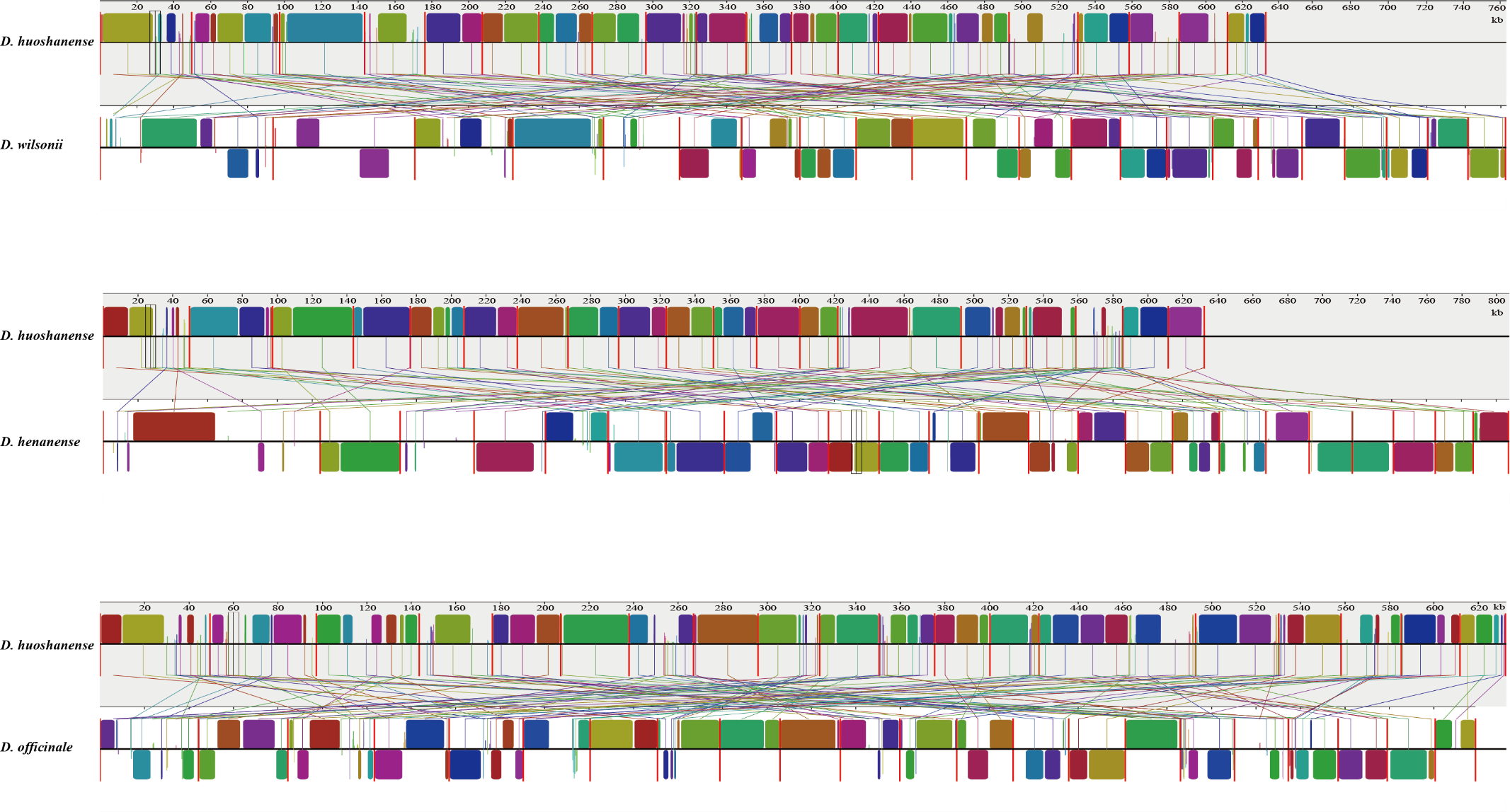


**Additional file 11: Figure S8.** Mauve alignments of *Dendrobium* mitogenomes. The mitogenome of *D*. *huoshanense* was selected as reference. Red lines represent boundaries between isoforms, the order was consistent with the genomic map of mitogenomes.
