# Supplementary material for: Mitochondrial genome comparison and phylogenetic analysis of Dendrobium (Orchidaceae) based on whole mitogenomes
Source: BMC Plant Biol. 2023 Nov 23;23:586. doi: 10.1186/s12870-023-04618-9 (PMC10666434; doi:10.1186/s12870-023-04618-9)
Supplement: Supplementary file 14 — Additional file 14: Figure S11. ML tree of 26 Dendrobium species inferred from whole mitogenomes excluded plastid-derived sequences. The number at each node was the percentage of bootstrap value (BS >50% was shown). [file 12870_2023_4618_MOESM14_ESM.docx]

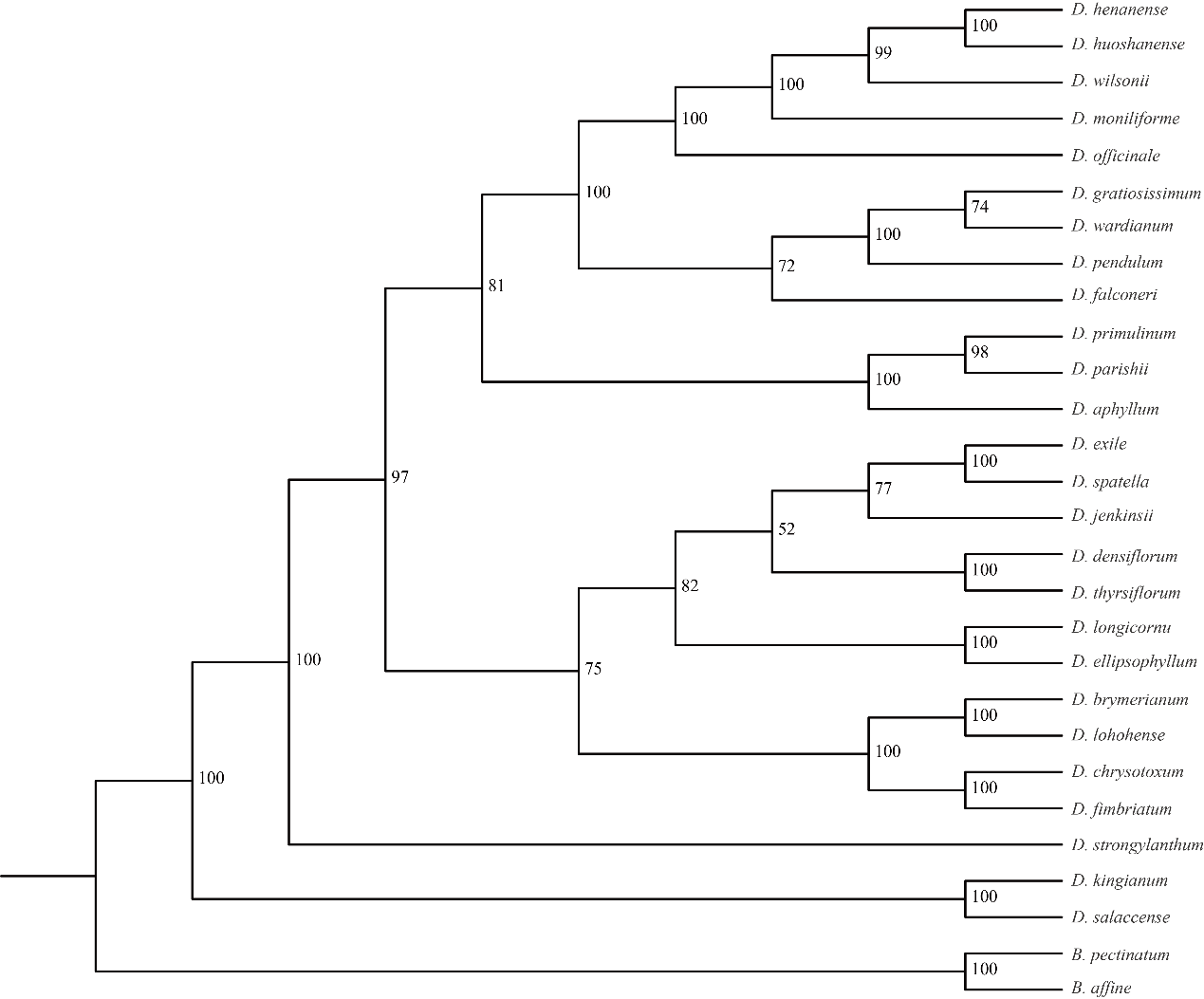


**Additional file 14: Figure S11.** ML tree of 26 *Dendrobium* species inferred from whole mitogenomes excluded plastid-derived sequences. The number at each node was the percentage of bootstrap value (BS >50% was shown).
