# Supplementary material for: Mitochondrial genome comparison and phylogenetic analysis of Dendrobium (Orchidaceae) based on whole mitogenomes
Source: BMC Plant Biol. 2023 Nov 23;23:586. doi: 10.1186/s12870-023-04618-9 (PMC10666434; doi:10.1186/s12870-023-04618-9)
Supplement: Supplementary file 15 — Additional file 15: Table S4. Sampling information of 28 species including two outgroups and 26 Dendrobium species. [file 12870_2023_4618_MOESM15_ESM.docx]

| Species | Chloroplast- GenBank accession | Voucher number | Mitochondrial- GenBank accession | Voucher number |
| --- | --- | --- | --- | --- |
| *D. henanense* | **LC727398** | WMT21_001 | / | WMT21_001 |
| *D. huoshanense* | LC193517 | **/** | / | LLD01_006 |
| *D. wilsonii* | LC490389 | **/** | / | WMT17_014 |
| *D. moniliforme* | LC490389 | **/** | / | WMT19_034 |
| *D. officinale* | LC348725 | **/** | / | ZSY01521 |
| *D. falconeri* | LC490395 | **/** | / | LLD14_208 |
| *D. wardianum* | LC490666 | **/** | / | LLD20_210 |
| *D. gratiosissimum* | LC490662 | **/** | / | LLD17_156 |
| *D. pendulum* | LC490698 | **/** | / | LLD26_232 |
| *D. primulinum* | LC490376 | **/** | / | WMT17_008 |
| *D. parishii* | LC193518 | **/** | / | WMT19_035 |
| *D. aphyllum* | LC490671 | **/** | / | LLD13_186 |
| *D. brymerianum* | LC192954 | **/** | / | WMT17_005 |
| *D. lohohense* | LC490668 | **/** | / | LLD22_237 |
| *D. chrysotoxum* | LC193517 | **/** | / | LLD25_256 |
| *D. fimbriatum* | LC193521 | **/** | / | WMT17_013 |
| *D. exile* | LC193517 | **/** | / | WMT17_011 |
| *D. spatella* | LC193511 | **/** | / | WMT19_032 |
| *D. jenkinsii* | LC193515 | **/** | / | WMT18_019 |
| *D. densiflorum* | NC_054181 | **/** | / | WMT19_026 |
| *D. thyrsiflorum* | LC528136 | **/** | / | WMT18_025 |
| *D. longicornu* | MN227146 | **/** | / | WMT18_023 |
| *D. ellipsophyllum* | LC193519 | **/** | / | WMT17_006 |
| *D. strongylanthum* | LC490685 | **/** | / | WMT18_017 |
| *D. salaccense* | LC193510 | **/** | / | WMT19_036 |
| *D. kingianum* | LC331062 | **/** | / | WMT19_041 |
| *B. affine* | LC556091 | **/** | / | WMT17_057 |
| *B. pectinatum* | LC556091 | **/** | / | WMT17_058 |

Table S4. Sampling information of 28 species including two outgroups and 26 *Dendrobium* species.
