# Supplementary material for: Mitochondrial genome comparison and phylogenetic analysis of Dendrobium (Orchidaceae) based on whole mitogenomes
Source: BMC Plant Biol. 2023 Nov 23;23:586. doi: 10.1186/s12870-023-04618-9 (PMC10666434; doi:10.1186/s12870-023-04618-9)
Supplement: Supplementary file 16 — Additional file 16: Table S5. Accession numbers of 48 angiosperm mitogenomes for local database establishment. [file 12870_2023_4618_MOESM16_ESM.docx]

Table S5. Accession numbers of 48 angiosperm mitogenomes for local database establishment.

| Taxonomy | Species | GenBank Accession Number |
| --- | --- | --- |
| Apiales | *Bupleurum falcatum* | KX887330 |
|  | *Daucus carota* | LNRQ01000010 |
| Asterales | *Codonopsis lanceolata* | MG775430 |
|  | *Lactuca serriola* | NC_042378 |
|  | *Platycodon grandiflorus* | KX887331 |
| Asparagales | *Gastrodia elata* | MF070084-MF070102 |
|  | *Phalaenopsis aphrodite* | MN366132- MN366175 |
|  | *Dendrobium officinale* | LC640134‐LC640155 |
|  | *Dendrobium huoshanense* | LC657527‐LC657545 |
|  | *Allium cepa* | NC_030100 |
|  | *Chlorophytum comosum* | MW411187 |
|  | *Crocus sativus* | OL804177 |
|  | *Hemerocallis citrina* | MZ726801-MZ726803 |
| Amborellales | *Amborella trichopoda* | KF754801-KF754803 |
| Brassicales | *Brassica juncea* | NC_016123 |
|  | *Carica papaya* | EU431224 |
|  | *Eruca vesicaria* | KF442616 |
|  | *Raphanus sativus* | AP018472 |
|  | *Turritis glabra* | LC325489 |
|  | *Arabidopsis thaliana* | JF729201 |
| Caryophyllales | *Beta vulgaris* | BA000009 |
| Cucurbitales | *Citrullus lanatus* | GQ856147 |
|  | *Cucurbita pepo* | GQ856148 |
| Fabales | *Acacia ligulata* | NC_040998 |
|  | *Ammopiptanthus mongolicus* | NC_039660 |
|  | *Glycine soja* | NC_039768 |
|  | *Leucaena trichandra* | NC_039738 |
|  | *Vicia faba* | KC189947 |
|  | *Vigna radiata* | AP014716 |
|  | *Pongamia pinnata* | NC_016742 |
| Lamiales | *Ajuga reptans* | NC_023103 |
|  | *Olea europaea* | MG372116 |
| Magnoliales | *Liriodendron tulipifera* | MK340747 |
| Malpighiales | *Hevea brasiliensis* | AP014526 |
|  | *Ricinus communis* | NC_015141 |
|  | *Salix sinopurpurea* | NC_029693 |
| Malvales | *Bombax ceiba* | NC_038052 |
|  | *Gossypium barbadense* | NC_028254 |
|  | *Hibiscus cannabinus* | NC_029855 |
|  | *Lagerstroemia indica* | NC_035616 |
| Ranunculales | *Nymphaea colorata* | NC_037468 |
| Rosales | *Cannabis sativa* | NC_029855 |
|  | *Malus hupehensis* | KR534606 |
|  | *Ziziphus jujuba* | NC_029809 |
| Solanales | *Capsicum annuum* | KJ865410 |
|  | *Nicotiana tabacum* | NC_006581 |
|  | *Lycopersicon esculentum* | MF034193 |
| Vitales | *Vitis vinifera* | FM179380 |
